# Appropriation for the Investigation of Inoculation against Yellow Fever

**Published:** 1887-04

**Authors:** 


### APPROPRIATION FOR THE INVESTIGATION OF INOCULATION AGAINST YELLOW FEVER.

At last something is to be done for investigating the claims of yellow fever inoculation. Notwithstanding, heretofore, utter disregard by the officials of the United States government of the facts in support of this protection from yellow fever, measures should have been adopted two years ago for investigating this matter of the greatest practical importance to a large portion of our population. But while millions have been spent in very questionable enterprises, a hue and cry has been raised in certain quarters against the outlay of a few thousand dollars for the verification of the prophylactic properties of yellow fever inoculation. It is supposed that such opposition may have had its effect in limiting the sundry civil appropriation bills to "a sum not exceeding ten thousand dollars for the purpose of investigating the merits of the methods practiced in Mexico and Brazil for preventing yellow fever by inoculation."

If the exploration of these regions, in which inoculation by different processes has been adopted under the supervision of Freire and Cremona, can be accomplished by competent observers for the amount set apart from the United States treasury, of course there could be no good and sufficient reason for expending more money. But if there is ground to believe the statements already brought to the attention of Congress in a memorial from the committee appointed for this purpose by the American Medical Association, at its meeting in St. Louis last year, then ten millions of dollars would not be an expenditure disproportionate to the great ends to be attained by this investigation. A member of the United States medical staff, whose salary is guaranteed outside of this appropriation, is considered by the *Medical News* as the only man in this country who possesses the requisite qualifications for the chief of this commission. But it is claimed that the investigation of the evidence as to the effects of inoculation will require much time, patience and tact by a man who can speak Spanish well and is familiar with the subject. The *News* thinks such a man might well be appointed from civil life, and says there are several physicians in the South who possess the requisite qualifications; but it strikes us that few medical men, whose position in the profession would entitle them to this appointment, can afford to cut loose from their business obligations and serve the public for such compensation as is provided in this niggardly appropriation. If the distinction of conferring a grand benefaction upon the citizens of this country is reckoned as a fit return for services rendered to the United States government, then we feel assured that whoever enters upon this work will receive his reward in the plaudits of a grateful people for his good work.

Members of the medical profession who have at heart the best interests of their fellow-men, and the advancement of science, should be selected for this investigation, and there should be no further delay in entering upon this highly important work.

The recent reports of cholera in South America indicate that a visit to that section would not be desirable at present; and, indeed, the yellow fever season has closed auspiciously in Brazil. But the opportunity for studying yellow fever inoculation in Mexico will be afforded as our summer advances, and when winter comes here operations may be transferred to Brazil, where it will then be the summer season.

